# Death in the sauna-vitality markers for heat exposure

**DOI:** 10.1007/s00414-021-02504-3

**Published:** 2021-01-14

**Authors:** Anja Wegner, Elke Doberentz, Burkhard Madea

**Affiliations:** Institute of Legal Medicine, University Hospital Bonn, Stiftsplatz 12, 53111 Bonn, Germany

**Keywords:** Sauna, Heat shock, Heat stroke, Pre-mortem temperature influences, Heat shock proteins, Aquaporins

## Abstract

In sauna-associated deaths, the vitality of heat exposure is of great importance. Two case reports address this. First, we present the case of a 77-year-old man who was found dead in the sauna of his family home. When found, the sauna door was closed, and the sauna indicated a temperature of 78 °C. The body had already begun to decay and was partially mummified when it was found. In the other case, a 73-year-old woman was found dead in the sauna by her husband. In this case, the sauna door was also closed. The sauna was still in operation at a temperature of approximately 70 °C. Epidermal detachments were found. In both autopsies and their follow-up examinations, there were no indications of a cause of death competing with heat shock. The expression of heat shock proteins in kidneys and lungs and the expression of aquaporin 3 in skin were investigated to detect pre-mortal temperature influences.

## Introduction

Coronary atherosclerosis or acute myocardial infarctions were the main underlying causes of death in 78% of natural deaths in the sauna in a Finnish study. However, natural deaths comprise only 46% of all deaths in the sauna, while 54% die of non-natural causes. In 36%, death was assumed to be due to effects of heat, heat stroke, or burns. Because of the high ambient temperature, sauna deaths show some peculiarities; for instance, the accelerated onset of putrefactive changes makes the correct diagnosis of cause and manner of death difficult. The diagnosis of vitality of heat exposure is of special importance because the cause of death is often a combination of preexisting diseases, especially heart diseases, and exposure to high temperature [1–3]. We report two cases of sauna-associated deaths.

## Case reports

### Case 1

In the first case, a 77-year-old man was found dead in the sauna of his family home. It was suspected that the body had been lying in the sauna for about 3 days. A craftsman had been unable to meet the owner of the house several times on the agreed date and alerted the emergency services. When the man was found, the sauna door was closed, and the sauna showed a temperature of 78 °C. The upper body was lying on the right side of the first bench with the legs stretched out in the middle of the sauna. The body had already begun to decay and the skin was partially mummified. The man was a recovered alcoholic, and no other previous illnesses were known.

**Fig. 1:**
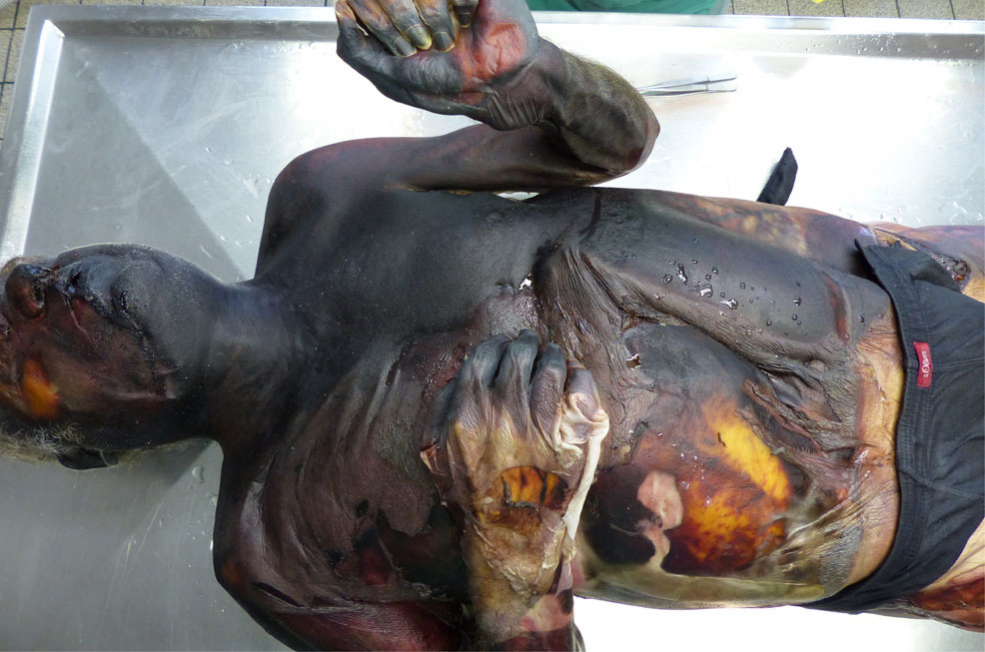
Almost completely mummified body at autopsy

The autopsy was conducted promptly. The man had a body weight of 73 kg and a body length of 179 cm. The autopsy revealed an advanced decayed body with partly leather-like dried skin (Fig. 1). The assessability of the internal organs was considerably limited because of the putrefaction and heat-related hardening and dehydration. Moderate general arteriosclerosis and coronary sclerosis, thickening of the left ventricular wall (approx. 2 cm) as a sign of arterial hypertension, cholecystolithiasis, and gastritis were found. The spleen was already liquefied. There were no indications for death-related violence. Histological examinations of the heart tissue showed a fatty degeneration of the myocardium and coronary sclerosis. Irregular enlargement of the myocytes and areas of myocardial fibrosis could also be detected.

### Case 2

In another case, a 73-year-old woman was found dead by her husband in a half-sitting position in the sauna in the basement of the apartment house. Here, also, the sauna door was closed. The sauna was still in operation at approximately 70 °C. The woman had only been in the sauna for about 35 min. When the emergency doctor arrived, the woman’s skin showed extensive detachments of the epidermis with blistering. The deceased had arterial hypertension and Parkinson’s disease. Additionally, she had complained about blood pressure problems several times previously.

At autopsy, extensive detachments of the epidermis with map-like desiccations and an incipient penetration of the venous network were shown. The woman had a body weight of 52 kg and a body length of 155 cm. Furthermore, calcification of the aortic valve was beginning to form, and low-grade aortic sclerosis and cholecystolithiasis were found. There were no indications for death-related violence. Histological examinations of the heart tissue showed an infiltration by adipose tissue, especially in the wall of the left ventricle. Some nuclei of the hypertrophic cardiac myocytes were enlarged. The coronary arteries showed a thickening of the intima.

No morphologically clear cause of death was found in either death. Chemicotoxicological investigations including determinations of blood alcohol concentration were conducted without appreciable findings. In neither case did histological examinations present organic diseases that could have caused death on their own. Heat damage in combination with a preexisting hypertensive cardiovascular disease was therefore considered the cause of death in both cases. To detect pre-mortem temperature influences on the body, the expression of heat shock proteins in kidneys and lungs and the expression of aquaporin 3 in skin were investigated.

## Material and methods

During forensic autopsies, tissue samples were taken of the lungs, kidneys, and skin for histological and immunohistochemical examination. The samples were fixed in 4% formalin. After fixation, they were embedded in paraffin wax and cut into slices (3–4 μm). Tissue of the lungs and kidneys was treated with hsp27, hsp60, and hsp70 antibodies and tissue of the skin with aquaporin 3 antibodies as well as with hematoxylin-eosin (H & E). In each staining procedure, skin tissue for hsp27 and 70, lung tissue for hsp60, and kidney tissue for aquaporin 3 were used as positive controls. In addition, one sample without the primary antibody and one without the secondary antibody were treated to serve as negative controls. Each slide was examined with a light microscope at × 200 magnification in 20 fields of view.

The immunohistochemical reaction of the tissue was then measured semiquantitatively on a four-degree scale according to Preuss et al. [4]. The number of positively reddish-stained structures/cells in relation to all examined structures/cells that were visible in each field of view was estimated as a percentage. For each analyzed organ structure and case, a mean value was calculated over all 20 fields of view and classified into the following grades: grade 0: no reaction; grade 1: < 30%, weak reaction; grade 2: < 60%, moderate reaction; and grade 3: up to 100%, intense reaction.

## Immunohistochemical findings

**Fig. 2:**
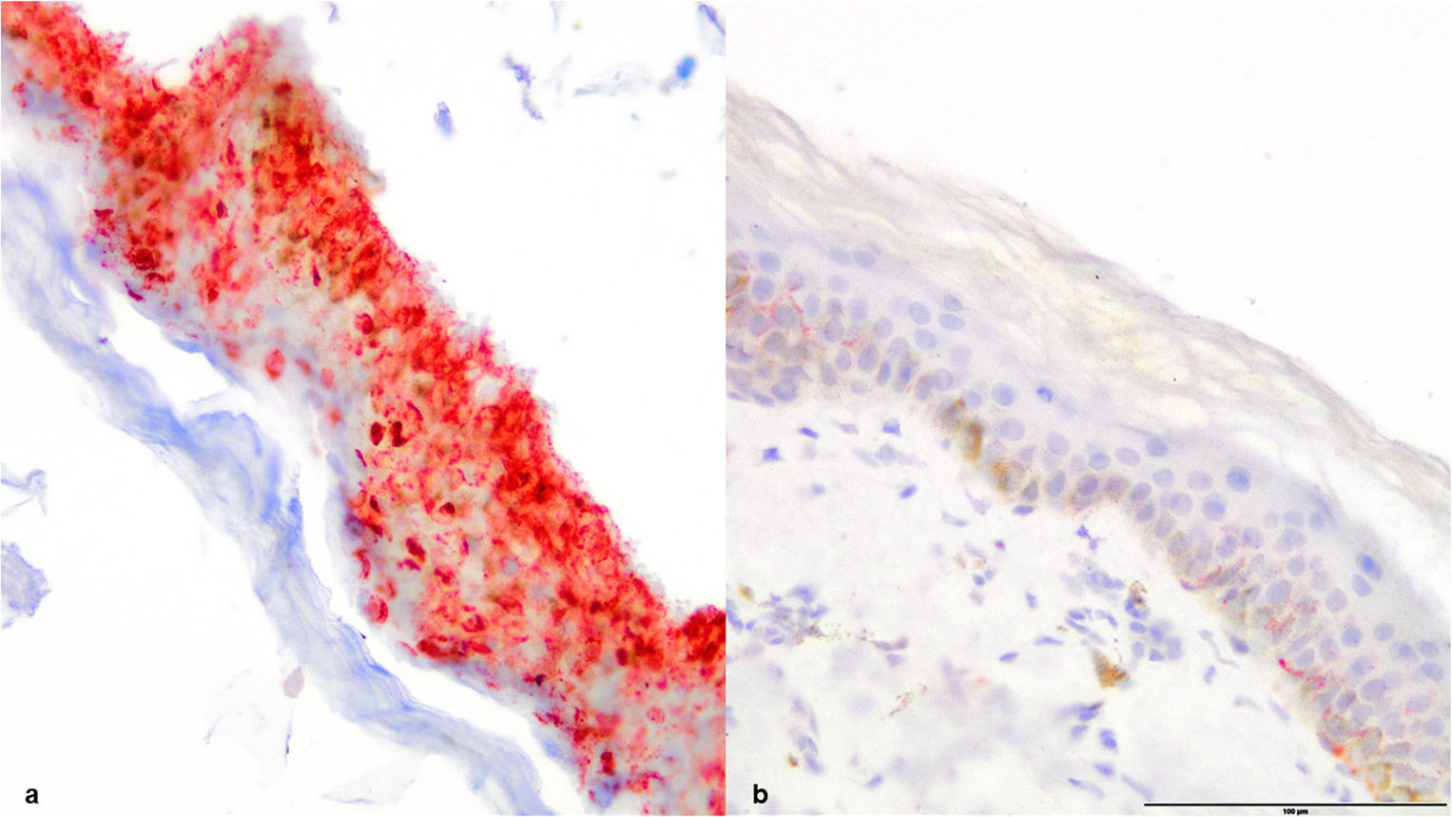
Skin, epidermis aquaporin 3 positive, (**a**) case 1, (**b**) control sample of skin, aquaporin 3 staining, × 200

In the first case immunohistochemically, no expression of heat shock proteins 27, 60, or 70 in the kidneys or lungs was detectable; however, intense aquaporin 3 expression in the epidermis (grade 4) was detectable. For comparison, there was just a weak reaction (grade 1) detectable in a control sample of skin that was not exposed to any heat stress (Fig. 2).

**Fig. 3:**
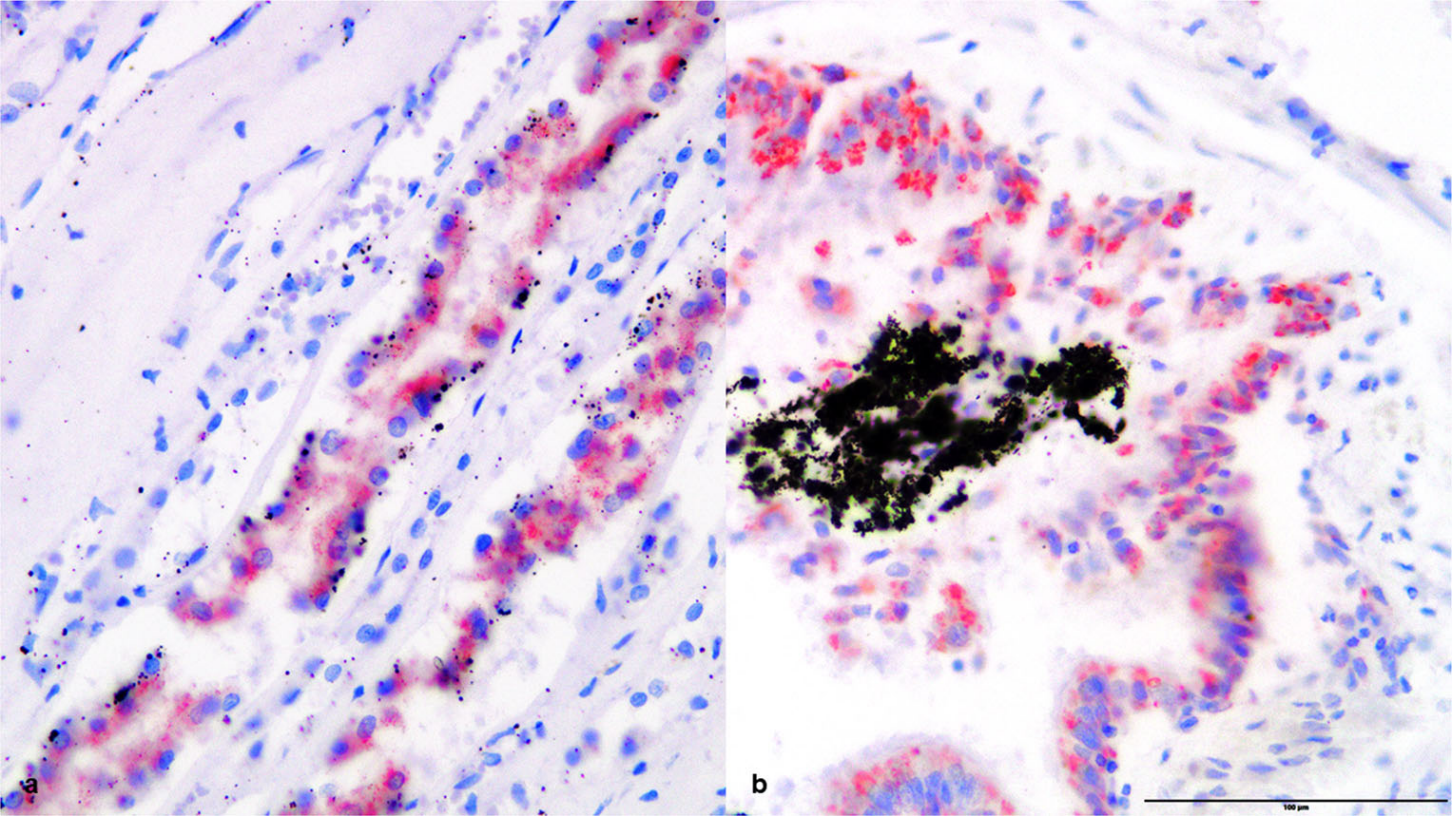
(**a**) Kidney tissue and (**b**) lung tissue, positive HSP27 staining, × 200

**Fig. 4:**
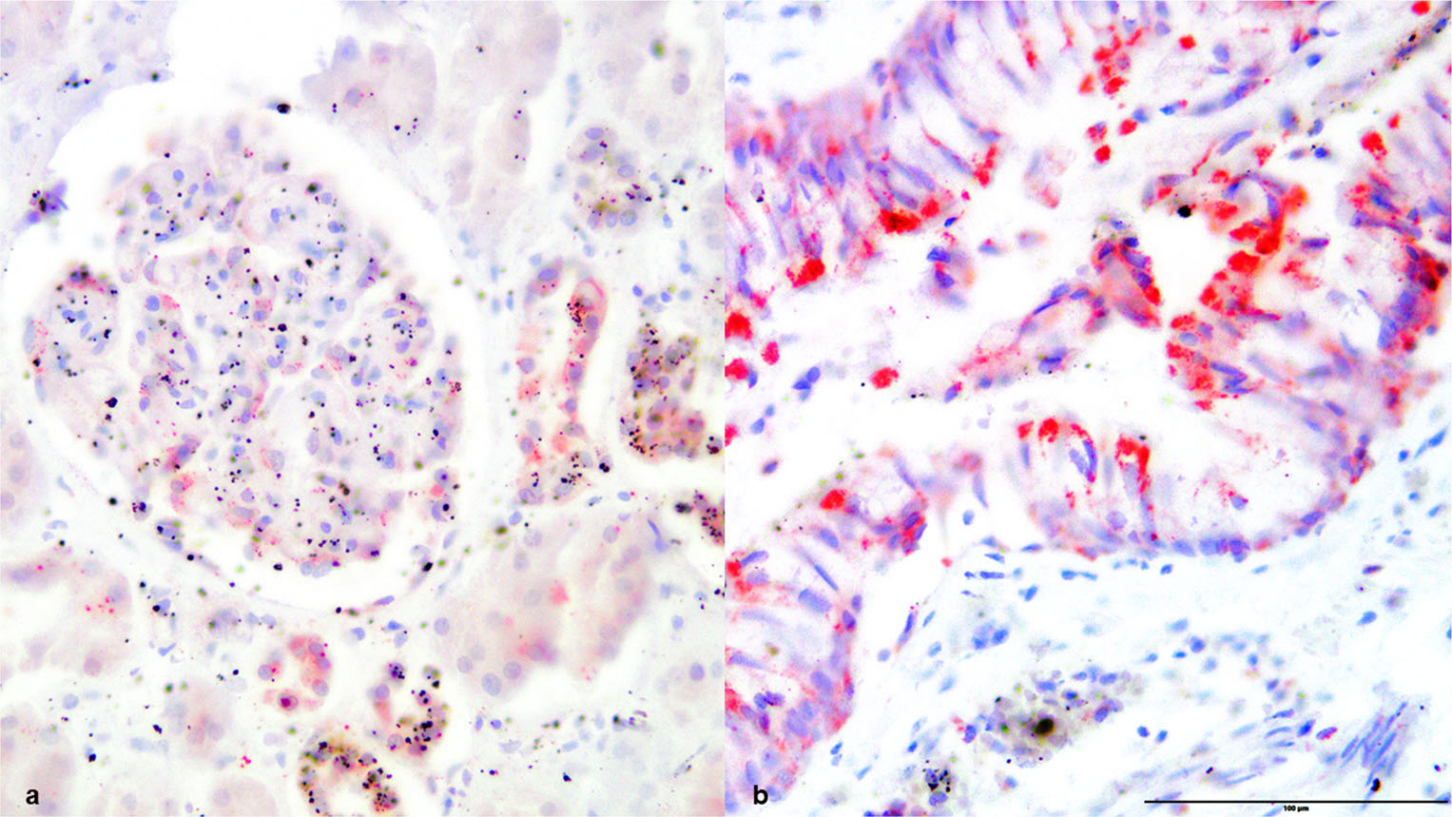
(**a**) Kidney tissue and (**b**) lung tissue, positive HSP60 staining, × 200

**Fig. 5:**
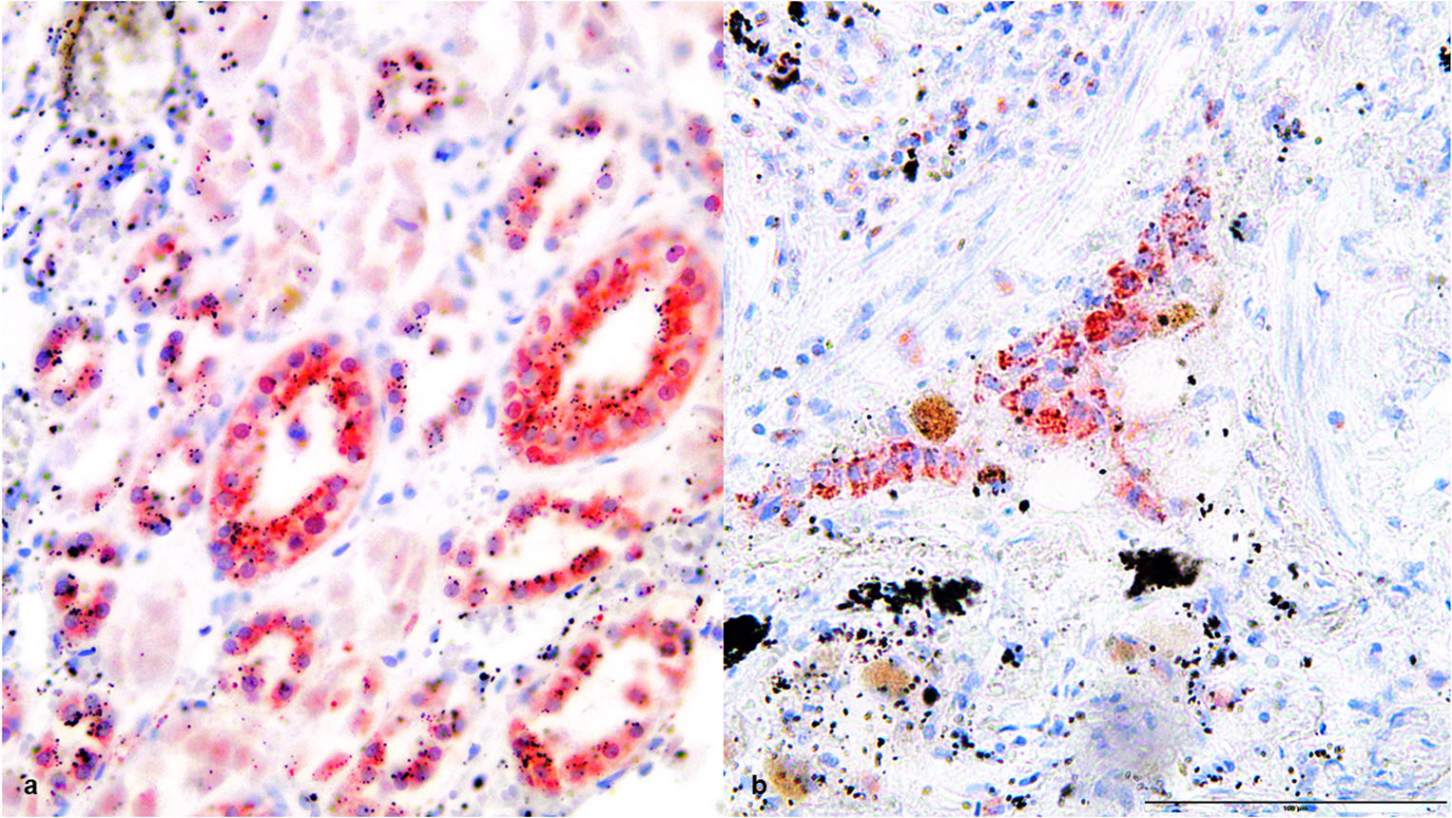
(**a**) Kidney tissue and (**b**) lung tissue, positive HSP70 staining, × 200

In the second case, an expression of heat shock proteins 27, 60, and 70 was found in the preserved lung and kidney tissue (Figs. 3, 4, and 5) as evidence for general overheating of the body. The epithelium of the central respiratory tract presented a third-grade reaction for hsp27, hsp60, and hsp70. In the kidney tissue, the tubules showed an intense reaction (grade 3) for hsp27 and hsp70. For hsp60, there was only a first-grade reaction detected in the tubules and the glomeruli.

## Discussion

Sauna visits are becoming increasingly popular. The thermal stimuli are said to have therapeutic effects on certain diseases (such as high blood pressure, asthma, and rheumatic diseases) [5]. However, sauna visits can also lead to systemic heat damage to the human body [6]. As a rule, however, deaths in the sauna tend to reveal rather unspecific findings during autopsy [7]. Additionally, the high temperatures accelerate changes in the body, which can make it difficult to determine whether the person died in the sauna or was taken there after death [1]. To detect pre-mortem temperature influences, therefore, the expression of heat shock proteins in kidneys and lungs and the expression of aquaporin 3 in skin should be investigated.

Heatstroke causes systemic overheating of the body with a body core temperature exceeding 40.5 °C and a consequent loss of consciousness up to coma [8]. It is therefore a disturbance of the body’s thermal regulation that results in an imbalance between the generation and dissipation of heat [9]. Water is constantly transported to the surface of the skin to evaporate there and thus cool the body. At ambient temperatures above body temperature, heat can only be given off in this way [10, 11]. If the body temperature cannot be lowered, this can lead to loss of water and electrolytes and to a circulatory decompensation with multi-organ failure [12, 13].

In both cases presented, death was apparently caused by an abnormally high external heat input, that is, high temperatures in the sauna (78 °C in the first case and 70 °C in the second case). Without the possibility of heat emission, heat stroke and acute circulatory decompensation occurred. It should be mentioned that without this preexisting hypertensive cardiovascular disease, circulatory decompensation in the sauna might not have occurred in any of the cases.

As both autopsies showed expected unspecific findings, the expression of aquaporin 3 in skin and of heat shock proteins 27, 60, and 70 in the kidney and lungs was examined to detect pre-mortal temperature influences (hyperthermia due to increased ambient temperature) on the body [14–16].

Aquaporin 3 belongs to the family of plasma membrane proteins and is expressed in human skin by epidermal keratinocytes. So far, there are known 13 aquaporins in humans. Together with aquaporin 1, it transports water and glycerol through the cell membrane and can be used to determine the age of wounds [17–23]. It is responsible for moisture regulation in the skin and therefore heat release [24]. In addition, increased aquaporin 3 expression was found in many types of cancer, for example, in lung cancer or colon cancer, and aquaporin 3 is necessary for skin tumor development [25].

In the first case, the intense expression of aquaporin 3 in the epidermis indicates thermal damage to the skin. Regarding the case history and the autopsy findings, there were no indications that the intense expression might have been increased due to other factors; for example, no malignancies were known or found at autopsy.

Heat shock proteins are produced in various organs during cellular stress, such as that caused by inflammation or hypoxia, but also when the body is exposed to heat, and have a protective effect on the cells [4, 26, 27]. It should be noted that despite vital heat exposure, a low or negative heat shock protein expression could be found, which can lead to false negative results. It is discussed that a gene polymorphism results in a lack of heat shock protein expression or an expression of ineffective or damaged heat shock protein, which could have an influence on the grading of the immunohistochemically visualized heat shock protein [16, 28]. This could possibly be the reason for the negative results in the first case.

As expected, the expression of aquaporin 3 and heat shock proteins was shown in both cases to reveal pre-mortem temperature influences on the body. Both aquaporin 3 and heat shock proteins are therefore suitable as vitality markers. Furthermore, the immunohistochemical representation of expression of heat shock proteins in kidney and lung tissue or of aquaporin 3 in skin can also be used to differentiate between vital and postmortem heat exposure.
